# Editorial: The Role of HMGB1 in Immunity

**DOI:** 10.3389/fimmu.2020.594253

**Published:** 2020-09-09

**Authors:** Myoungsun Son, Betty Diamond, Jeon-Soo Shin

**Affiliations:** ^1^Center for Autoimmune Musculoskeletal and Hematopoietic Diseases, Institute of Molecular Medicine, The Feinstein Institutes for Medical Research, Manhasset, NY, United States; ^2^Donald and Barbara Zucker School of Medicine at Hofstra/Northwell, Hempstead, NY, United States; ^3^Department of Microbiology, Yonsei University College of Medicine, Seoul, South Korea; ^4^Institute for Immunology and Immunological Diseases, Yonsei University College of Medicine, Seoul, South Korea; ^5^Center for Nanomedicine, Institute for Basic Science, Yonsei University, Seoul, South Korea

**Keywords:** HMGB1, secretion, inflammation, sepsis, cancer, immune function, potential therapeutics

High mobility group box 1 (HMGB1) is an evolutionarily conserved nuclear protein that can be released by almost all cell types. Scientists have uncovered a variety of molecular mechanisms by which HMGB1 in both immune and non-immune cells modulates the nature and magnitude of immune responses (1–3). In recent years, HMGB1-targeted therapies have been exploited in multiple preclinical studies of inflammatory conditions and there is robust clinical evidence for HMGB1 levels as a potential biomarker for early prediction or progression of various diseases. However, it is not presently possible to specifically target HMGB1 in any clinical setting. A significant obstacle to developing therapeutics lies in gaps in knowledge of the post-translational modification of HMGB1 as well as the timing and type of microenvironments to which HMGB1 is exposed.

This Research Topic provides a comprehensive overview of current understanding of the contribution of HMGB1 to various diseases and HMGB1 specific therapeutics. Nine articles are included: five original articles, three review articles, and one mini-review. The authors invited the scientific contributors to this collection based on their unique and pioneering discoveries on the role of HMGB1 in physiological and pathological conditions including: (i) HMGB1-related immune functions (ii) Post-translational modification and secretion mechanisms of HMGB1 (iii) Molecular pathways activated by HMGB1 in acute lung injury, lupus, cancers, and other diseases (iv) Agents to modulate HMGB1 function.

## HMGB1-related Immune Functions

While many researchers have focused on HMGB1 as an inflammatory mediator that prolongs various inflammatory diseases, another aspect of HMGB1, which is related to its role in tissue healing and regeneration, is being highlighted (4, 5). Yamashiro et al. describe the potential tolerogenic role of HMGB1 in periodontal disease progress, including the cause of inflammation and, conversely, regeneration of periodontal tissue. Further studies are needed regarding HMGB1 isoforms and their receptors that play major roles in the oral cavity to open up opportunities for therapeutics.

Serum HMGB1 is elevated in systemic lupus erythematosus (SLE) patients, and it correlates with disease activity (6). There are several preclinical studies of HMGB1-specific antagonists in experimental lupus models showing inconsistent results. Liu et al. provide a mini-review about the role of HMGB1 in SLE disease phenotypes and a novel agent forcing anti-inflammatory macrophages polarization.

In the tumor microenvironment, HMGB1 has a protective role in cancer immunity during the early stage of disease. In contrast, sustained HMGB1 recruits immunosuppressive myeloid-derived suppressor cells and regulatory T cells during tumor progression (7). Soloff et al. provide insight into how HMGB1 impacts the microenvironment of malignant pleural effusions (MPEs). The level of HMGB1 was inversely correlated to the diversity of γδ T cells in MPE. The authors suggest some novel therapeutic strategies for targeted HMGB1-neutralization and its usage in pleural effusions.

## Post-Translational Modification and Secretion Mechanisms of HMGB1

The dynamics of HMGB1 oxidation in health and disease are unknown. Ferrara et al. confirmed our understanding of functions of HMGB1 redox isoforms using novel applications of *in vivo*-based assay. They demonstrate that the redox state of HMGB1 is controlled at both tissue and cell levels, suggesting that HMGB1 oxidation is a spatially regulated process. Kwak et al. provide an overview of the protein secretion mechanisms. The authors highlight the importance of multiple post-translational modifications and the redox biology of HMGB1, focusing on the vital role of HMGB1 oxidation in its secretion.

## Molecular Pathways by HMGB1 in Human Diseases

Sepsis is a life-threatening inflammatory condition with no known cure. HMGB1 is a critical mediator of acute and chronic inflammation in sepsis caused by endotoxin (8). Li W. et al. assess a novel mechanism through which hepatocytes secrete HMGB1 following LPS stimulation that is relevant to sepsis pathogenesis and inflammatory diseases of the liver. The cytoplasmic translocation and later release of HMGB1 from hepatocytes are mediated by a TLR4, Caspase-11, and Gasdermin D-dependent mechanism. HMGB1 is secreted in exosomes. Kim et al. demonstrate the anti-inflammatory effect of sulfatide in suppressing the secretion of HMGB1 and disrupting lipid rafts following LPS stimulation. They suggest that sulfatide is a potential therapeutic agent against sepsis. Li R. et al. explore how HMGB1/PI3K/Akt/mTOR signaling participates in acute lung injury and acute respiratory distress syndrome which are characterized by persistent hypoxemia, disruption of the alveolar-capillary barrier, and widespread inflammation in the lung.

## Agents to Modulate HMGB1 Function

As mentioned above, HMGB1 antagonists have achieved therapeutic success in a broad set of preclinical inflammatory disease animal models. Yang et al. summarize recent advances in the understanding of HMGB1 as a pro-inflammatory molecule.

Collectively, these articles provide information for other researchers in the field that will eventually help develop novel therapeutic approaches to regulate the function of HMGB1 for the benefit of patients. The next step should be to translate these preclinical studies into clinical settings. Many inflammatory diseases, including the current pandemic COVID-19, are characterized by increased circulating HMGB1 levels (9). HMGB1 possibly plays a role in the increased risk for severe outcomes in COVID-19 patients with inflammatory comorbidities. Overall, HMGB1 is relevant in many diseases and research on HMGB1 can benefit all fields of medicine.

## Author Contributions

MS wrote the manuscript. BD and J-SS contributed to the elaboration of the manuscript. All authors have approved it for publication.

## Conflict of Interest

The authors declare that the research was conducted in the absence of any commercial or financial relationships that could be construed as a potential conflict of interest.
